# Vacuum Assisted Closure Therapy versus Standard Wound Therapy for Open Musculoskeletal Injuries

**DOI:** 10.1155/2013/245940

**Published:** 2013-06-26

**Authors:** Kushagra Sinha, Vijendra D. Chauhan, Rajesh Maheshwari, Neena Chauhan, Manu Rajan, Atul Agrawal

**Affiliations:** ^1^Department of Orthopaedics, Himalayan Institute of Medical Sciences, Dehradun, Uttarakhand 248140, India; ^2^Himalayan Institute of Medical Sciences, Dehradun, Uttarakhand 248140, India; ^3^Department of Pathology, Himalayan Institute of Medical Sciences, Dehradun, Uttarakhand 248140, India; ^4^Department of Plastic Surgery, Himalayan Institute of Medical Sciences, Dehradun, Uttarakhand 248140, India

## Abstract

*Background*. This study was performed to evaluate the results of vacuum assisted wound therapy in patients with open musculoskeletal injuries. *Study Design and Setting*. Prospective, randomized, and interventional at tertiary care hospital, from 2011 to 2012. *Materials and Methods*. 30 patients of open musculoskeletal injuries underwent randomized trial of vacuum assisted closure therapy versus standard wound therapy around the upper limb and lower limb. Mean patient age was 39 ± 18 years (range, 18 to 76 years). Necrotic tissues were debrided before applying VAC therapy. Dressings were changed every 3 or 4 days. For standard wound therapy, debridement followed by daily dressings was done. *Data Management and Statistical Analysis*. The results obtained were subjected to statistical analysis. *Results*. The size of soft tissue defects reduced more than 5 mm to 25 mm after VAC (mean decrease of 26.66%), whereas in standard wound therapy, reduction in wound size was less than 5 mm. A free flap was needed to cover exposed bone and tendon in one case in standard wound therapy group. No major complication occurred that was directly attributable to treatment. *Conclusion*. Vacuum assisted wound therapy was found to facilitate the rapid formation of healthy granulation tissue on open wounds in the upper limb and lower limb, thus to shorten healing time and minimize secondary soft tissue defect coverage procedures.

## 1. Introduction

Wound healing is a complex and dynamic process that includes an immediate sequence of cell migration leading to repair and closure. This sequence begins with removal of debris, control of infection, clearance of inflammation, angiogenesis, deposition of granulation tissue, contraction, remodelling of the connective tissue matrix, and maturation. When wound fails to undergo this sequence of events, a chronic open wound without anatomical or functional integrity results [1].

High-energy open fractures require both skeletal stability and adequate soft tissue coverage. In such injuries, debridement of all nonviable tissue can produce significant soft-tissue defects precluding healing through primary closures, delayed primary closures, or secondary intention [2]. Various surgical methods have been developed to obtain coverage in these difficult situations. These include skin grafts, local rotation flaps, and myocutaneous or fasciocutaneous tissue transfers. Although skin grafts are readily obtainable, they are dependent on the vascularity of its recipient bed and may be contraindicated when exposed bone, cartilage, tendons, or surgical implants exist [3]. In such situation, a local rotation flap may be needed. When the soft tissue defect prevents local coverage [4], free tissue transfers are usually required, but the transfer may produce donor site morbidity and require late revisions due to the size of the muscle flap [5].

Although nonoperative modalities, such as hyperbaric oxygen, have been used to enhance wound coverage, these devices may not be available to all patients and may not be adequate for use in patients presenting with high-energy injuries due to edema, retraction of the skin and soft tissue, wound size, or loss of available local coverage [6]. Attempts have been made to identify an alternative treatment of wound management in these patients.

Clinically, chronic wounds may be associated with pressure sore, trauma, venous insufficiency, diabetes, vascular disease, or prolonged immobilization. The treatment of chronic, open wounds is variable and costly, demanding lengthy hospital stays or specialized home care requiring skilled nursing and costly supplies. Rapid healing of chronic wounds could result in decreased hospitalization and an earlier return of function. A method that improves the healing process could greatly decrease the risk of infection, amputation, and length of hospital stay and result in an estimated potential annual savings of billions of rupees of healthcare cost [1].

Initially developed in the early 1990s, for the management of large, chronically infected wounds that could not be closed in extremely debilitated patients, the use of vacuum-assisted closure (VAC) has been more recently used in the treatment of traumatic wounds [7].

The purpose of this study is to evaluate the results of this therapy for the management of patients presenting with open musculoskeletal injuries.

## 2. Materials and Methods

The study was conducted on 30 patients in the Department of Orthopaedics, Himalayan Institute of Medical Sciences, over a period of 12 months, after obtaining the permission from institutional ethical committee and taking informed and written consents from the patients.

All patients above 18 years of age with open musculoskeletal injuries in extremities that required coverage procedures were included in the study. However, patients with preexisting osteomyelitis in the wounds, neurovascular deficit in the injured limb, diabetics, malignancy, and peripheral vascular disease were excluded from the study.

The patients were prospectively randomized into one of the two treatment groups receiving either the vacuum assisted closure therapy or standard saline-wet-to-moist wound care. Files were marked with red (vacuum assisted closure therapy) or yellow (saline-wet-to-moist dressings) labels on the inside panel and were randomly organized. A file was randomly picked for each wound with the treatment determined by the label colour.

Participation in the study did not deviate from the standard care of the acute wound. All patients for wound management were subjected to

1. standard radiological assessment of the injured wound,
2. routine haematological investigation, for example, complete blood count, ESR, blood sugar, HIV and HbsAg, gram stain and culture,
3. all patients were supplemented with standard nutritional supplements, including zinc and multivitamin daily.

### 2.1. Vacuum Assisted Wound Therapy Procedure

#### 2.1.1. Wound Preparation

Any dressings from the wound were removed and discarded. A culture swab for microbiology was taken before wound irrigation with normal saline. Necrotic tissues were surgically removed (surgical debridement), and adequate haemostasis was achieved. Prior to application of the drape, it was essential to prepare the peri-wound skin and ensure that it was dry.

#### 2.1.2. Placement of Foam

**Figure 1:**
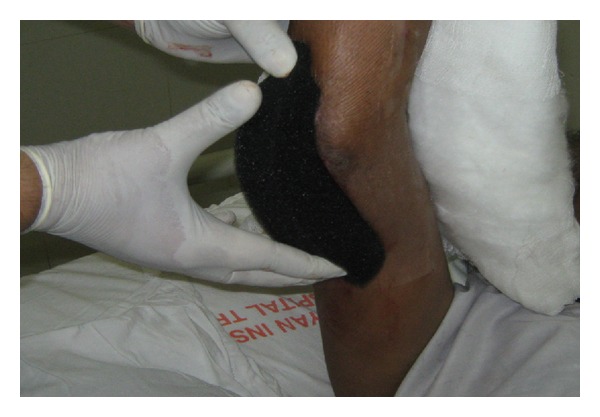
Foam was cut according to wound size.

Sterile, open-pore foam (35 ppi density and 33 mm thick) dressing was gently placed into the wound cavity. Open-pore foams are polyurethane with 400–600 microns size having hydrophobic open cell structured network. Such sizes of pores are most effective at transmitting mechanical forces across the wound and provide an even distribution of negative pressure over the entire wound bed to aid in wound healing (Figure 1).

#### 2.1.3. Sealing with Drapes

**Figure 2:**
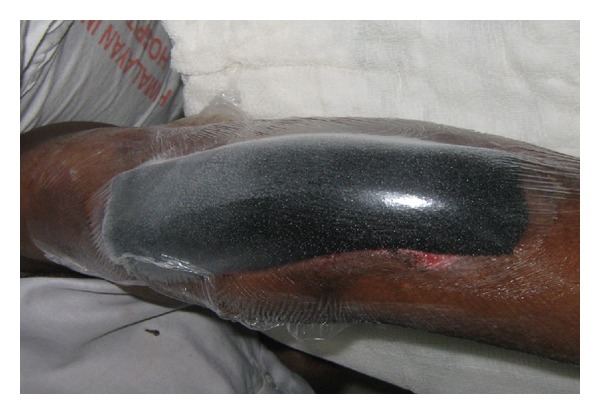
Sterile drape was applied covering the foam and 2-3 cm of the surrounding skin.

**Figure 3:**
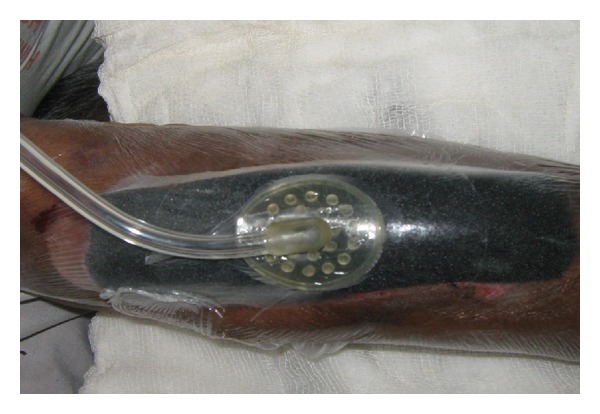
The connecting tube was applied after making a small opening (3-4 mm) on the drape.

The site was then sealed with an adhesive drape covering the foam and tubing and at least three to five centimetres of surrounding healthy tissue to ensure a seal (Figures 2 and 3).

#### 2.1.4. The Application of Negative Pressure

**Figure 4:**
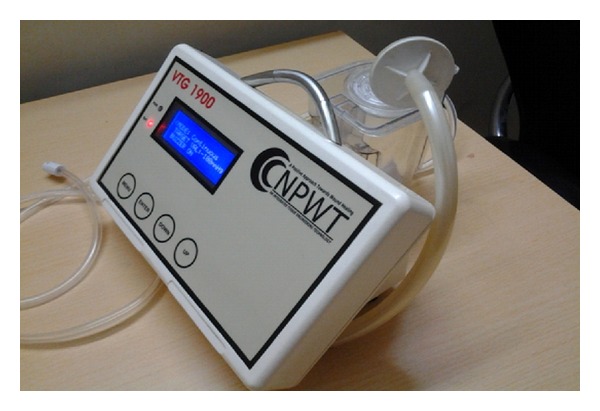
The connecting tube was connected to the negative pressure wound therapy.

Controlled pressure was uniformly applied to all tissues on the inner surface of the wound. The pump delivered an intermittent negative pressure of −125 mmHg. The cycle was of seven minutes in which pump was on for five minutes and off for two minutes (Figure 4).

The dressings were changed on the fourth day.

### 2.2. Saline-Wet-to-Moist Group Procedure

Wound preparation—any dressings from the wound was removed and discarded. A culture swab for microbiology was taken before wound irrigation with normal saline. Surface slough or necrotic tissue was surgically removed (surgical debridement), and adequate haemostasis was achieved. 

Daily dressings by conventional methods, that is, cleaning with hydrogen peroxide and normal saline and dressing the wound with povidone iodine (5%) and saline-soaked gauze was done and wound examined daily.

Resident who had measured the wounds was not involved in the daily care of the study patients. It was not mentioned to which treatment group the patient was assigned. This blinding arrangement ensured that the person evaluating the wound and collecting data initially at day zero and whenever dressings were subsequently changed had seen the wound only after all dressings, supplies, and equipment were removed from the patient and the room. He took photographs and measured the wound by Vernier Caliper and transparent O.H.P sheets (overhead projector sheet).

The resident doctor also clinically assessed the wounds for signs of infection and obtained 4–6 mm punch biopsy samples for histology and culture. Biopsies were obtained from the four corners and the most “healthy” portion of the wound bed. Samples were taken on day zero, day four, and day eight. The presence of drainage, edema, erythema, exposed bone, or exposed tendon wasdocumented. Any complications associated with vacuum assisted closure therapy were also documented. Such measurements and findings were recorded on day zero, day four, and day eight in both the groups.

The wounds were also evaluated by plastic surgeon on day one and on day eight to assess the nature of surgical procedure to be adopted to cover the wound. 

The pathologist noted and quantified the presence of inflammatory cells, bacteria, arterioles, proliferative fibroblasts, excessive collagen formation, and fibrosis in the biopsy samples.

### 2.3. Data Management and Statistical Analysis

The results obtained were subjected to statistical analysis which was done by using statistical software SPSS-version 19. Normality of data was checked by Kolmogorov-Smirnov test for unpaired *t*-test. Quantitative data was expressed in terms of mean ± SD. Categorical data was analysed by Chi-square and Wilcoxon signed ranks test.

For Wilcoxon signed ranks test, the evaluation of histological parameters (Inflammatory cells, proliferative fibroblasts, collagen formation, and fibrosis) was ranked as absent—0, mild—1, moderate—2, and severe—3. 

## 3. Results

Mean patient age was 39 ± 18 years (range, 18 to 76 years). All patients had suffered an acute trauma. Road traffic accident was found to be most common cause with 22 (73.33%) patients, followed by machinery injury in 5 (16.66%) patients and 3 (10%) patients had a fall from height. According to Gustilo Anderson classification, out of 30 patients, 16 patients had grade IIIb injury, 7 had grade IIIc injury, 3 had IIIa injury, and 4 had grade II injury.

*Decrease in Wound Size.* There was significant decrease in wound size from day zero to day eight in VAC group in comparison to saline-wet-to-moist group as shown in Tables 1 and 2.

*Bacterial Growth.* There was significant decrease in the bacterial growth in the VAC group as compared to saline-wet-to-moist group, as shown in Table 3. 

On histological comparison too, there was a statistical difference between the VAC group and saline-wet-to-moist group, *P* value being less than 0.05 by using Wilcoxon signed rank test between the findings from day zero to day eighth as shown in Table 4.

**Figure 5:**
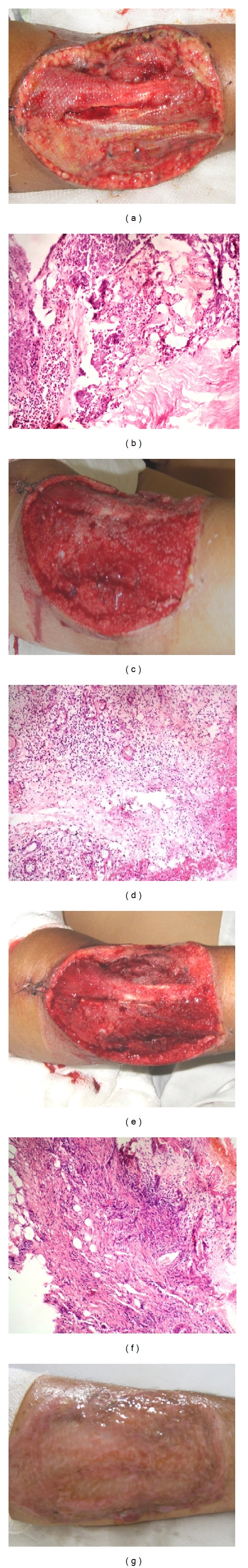
(a) Day 0: wound size, 146 × 135 mm. (b) Day 0: photomicrograph number 1-H and E stained section (100x) shows: dense neutrophilic exudates on the surface of wound. (c) Day 4: wound size, 130 × 120 mm. (d) Day 4: photomicrograph number 2-H and E stained section (100x) shows: fibrinous exhudate on the surface and base of ulcer is formed by moderately inflamed granulation tissue. (e) Day 8: wound size, 130 × 117 mm. (f) Day 8: photomicrograph number 3-H and E stained section (100x) shows: many newly formed blood vessels and dense fibro collagenous tissue. (g) SSG uptake seen.

*Case No. 1*. In an 18-year-male old a case of open grade IIIb fracture both bone right forearm (middle 3rd), wound was present over anterior aspect of forearm. After thorough debridement and fracture fixation VAC was applied (see Figure 5). 

**Figure 6:**
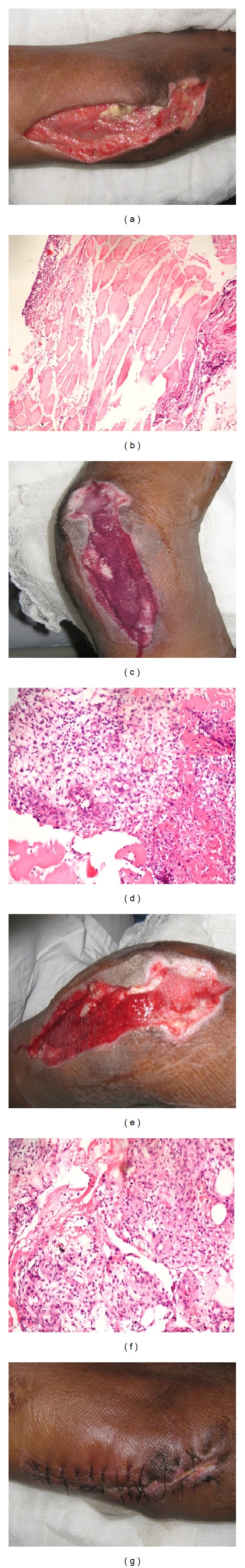
(a) Day 0: wound size, 146 × 61 mm. (b) Day 0: photomicrograph number 1-H and E stained section (100x) shows: thick neutrophillic exhudate on the surface and skeletal muscle bundles. (c) Day 4: wound size, 141 × 51 mm. (d) Day 4: photomicrograph number 2-H and E stained section (100x) shows: Inflammed granulation tissue with little exhudate on the surface. (e) Day 8: Wound size, 135 × 51 mm. (f) Day 8: photomicrograph number 3-H and E stained section (100x) shows: healthy granulation tissue without any exhudate. (g) Secondary closure done.

*Case No. 2*. In a 57-year-male old sustained injury open grade IIIa olecranon, debridement was done, and VAC was applied (see Figure 6).

## 4. Discussion

Healing is an intricate, interdependent process that involves complex interactions between cells, the cellular microenvironment, biochemical mediators, and extracellular matrix molecules that usually results in a functional restoration of the injured tissue [8, 9]. The goals of wound healing are to minimize blood loss, replace any defect with new tissue (granulation tissue followed by scar tissue), and restore an intact epithelial barrier as rapidly as possible.

The rate of wound healing is limited by the available vascular supply and the rate of formation of new capillaries and matrix molecules [10]. These events are heavily influenced by locally acting growth factors that affect various processes including proliferation, angiogenesis, chemotaxis and migration, gene expression, proteinases, and protein production [8, 11–14]. Disruption of any of these factors may adversely affect the healing process, resulting in a chronic or nonhealing wound.

Blood flow increases and bacterial colonization of wound tissues decreases following the application of subatmospheric pressure to wounds [7]. Any increase in circulation and oxygenation to compromised or damaged tissue enhances the resistance to infection [15]. Successful, spontaneous healing and healing following surgical intervention are correlated with tissue bacterial counts of less than 10^5^ organisms per gram of tissue [16]. Higher levels uniformly interfere in wound healing. Increase in local tissue oxygen levels reduce or eliminate the growth of anaerobic organisms, which have been correlated to decreased healing rates [17, 18]. Additionally, the increased flow should make greater amounts of oxygen available to neutrophils for the oxidative bursts that kill bacteria [19].

Our study showed that in VAC group after day 4, there were 20% of patients who had no bacterial growth, and on day 8 there were 60% of patients who had no bacterial growth, whereas in saline-wet-to-moist patients only 20% of patients had no bacterial growth on the 8th day. There have been similar studies by Morykwas and Argenta [7], Banwell et al. [20], and Morykwas et al. [21] which showed clearance of bacteria from infected wounds using VAC therapy.

On the other hand, Weed et al. while quantifying bacterial bioburden during negative pressure wound therapy concluded with serial quantitative cultures that there is no consistent bacterial clearance with the VAC therapy, and the bacterial growth remained in the range of 10^4^–10^6^ [22]. 

Thomas first postulated that application of mechanical stress would result in angiogenesis and tissue growth. Unlike sutures or tension devices, the VAC can exert a uniform force at each individual point on the edge of the wound drawing it toward the centre of the defect by mechanically stretching the cells when negative pressure is applied [23]. This allows the VAC to move distensible soft tissue, similar to expanders, towards the centre of the wound, thereby decreasing the actual size of the wound [24].

Our study showed a decrease in size of 1 to 4.9 mm in 26.66% of patients in VAC group whereas 93.33% in control group from day 0 to day 8. A decrease in size of 10 to 19.9 mm was seen in 46.66% of patients of VAC group and only 6.66% in control group. A decrease in size of more than 25 mm was seen in 13.33% in VAC group.

There have been similar studies by Joseph et al. [1], Morykwas and Argenta [7], and Morykwas et al. [21] which showed that VAC proved effective in shrinking the widths of wound over time compared to standard wound dressings.

Our study showed that VAC increases the vascularity and the increase in rate of granulation tissue formation compared to standard wound dressing. Histologically, VAC patients showed angiogenesis and healthy tissue growth as compared to the inflammation and fibrosis seen in standard wound dressing. Inflammation had increased in those treated with standard wound therapy and decreased in those patients treated with VAC.

The highly significant increase in the rate of granulation tissue formation of subatmospheric pressure-treated wound is postulated to be due to transmission of the uniformly applied force to the tissues on the periphery of the wound. These forces both recruit tissues through viscoelastic flow and promote granulation tissue formation. Currently, the Ilizarov technique and soft tissue expanders both apply mechanical stress to tissues to increase mitotic rates [25, 26].

Standard wound dressings adhere to devitalized tissue and within four to six hours the gauze can be removed, along with the tissue, as a form of mechanical debridement. This method of wound care has been criticized for removing viable tissue as well as nonviable tissue and being traumatic to granulation tissue and to new epithelial cells [27]. 

There is very little literature available especially on compound injuries using VAC. Open musculoskeletal injuries have very high incidences of nonunion and infection [28]; they require urgent irrigation and debridement. As wounds are frequently left open and require repeated debridement, resulting in large soft tissue defects, early coverage of exposed bone, tendons, and neurovascular structures is crucial. This is to decrease the risk of infection, nonunion, and further tissue loss. We believe that VAC therapy can be effective to overcome all the aforementioned problems.

The daily rental charges for a VAC machine and consumables are significant. This has discouraged many from using the system. However, there have been some reports showing that the increased healing times and downgrading of required operations correlate to decreased overall costs of care. The dressing should also enable larger wounds to be treated in the community with minimal nursing care impact. This would free up hospital beds permitting faster healing of operative patients and preventing waiting list buildup [29].

VAC therapy can be regarded as a method that combines the benefit of both open and closed treatment and adheres to DeBakey's principles of being short, safe, and simple. It has been shown to work and be beneficial to wound healing. VAC therapy is not the answer for all wounds; however, it can make a significant difference in many cases. VAC is most useful in difficult cavity or highly exudative wounds. VAC is a useful tool in moving a wound to a point where more traditional dressings or more simple surgical reconstructive methods can be used. As such it is a well deserved, although at present pragmatic addition to the wound healing armamentarium and the reconstructive ladder [30].

## 5. Conclusion

Vacuum assisted closure therapy appears to be a viable adjunct for the treatment of open musculoskeletal injuries. Application of subatmospheric pressure after the initial debridement to the wounds results in an increase in local functional blood perfusion, an accelerated rate of granulation tissue formation, and decrease in tissue bacterial levels. Although traditional soft tissue reconstruction may still be required to obtain adequate coverage, the use of this device appears to decrease their need overall.

## Figures and Tables

**Table 1:** Decrease in wound size from day 0 to day 8.

Measurements (mm)	VAC (*n* = 15)	Saline-wet-to-moist (*n* = 15)
1–4.9	4 (26.66%)	14 (93.33%)
5–9.9	1 (6.66%)	0
10–14.9	4 (26.66%)	1 (6.66%)
15–19.9	3 (20%)	0
20–24.9	1 (6.66%)	0
>25	2 (13.33%)	0

Chi Square = 14.4, d.f = 5, *P* = 0.013.

**Table 2:** Mean wound size difference between VAC and saline wet to moist on day 8.

	VAC (*n* = 15)	Saline wet to moist (*n* = 15)	*P* value	95% CI
Mean difference (mm)	13.24 ± 8.48	3.02 ± 2.90	0.0001	11.053 to 15.327

**Table 3:** Bacterial growth (*n* = 30).

Bacterial growth	VAC patients (*n* = 15)			Saline wet to moist (*n* = 15)		
	Day 0	Day 4	Day 8	Day 0	Day 4	Day 8
Present	15 (100%)	12 (80%)	6 (40%)	15 (100%)	15 (100%)	12 (80%)
Absent	0	3 (20%)	9 (60%)	0	0	3 (20%)

**Table 4:** Comparison of histological parameters from day 0 to day 8 by Wilcoxon signed ranks test.

Stages of wound healing	Saline wet to moist				VAC			
	Positive	Negative	Equal	*P* value	Positive	Negative	Equal	*P* value
Inflammatory cells	1	10	4	0.001	1	12	2	0.003
Proliferative fibroblasts	13	0	2	0.001	15	0	0	0.001
Collagen formation	14	0	1	0.001	15	0	0	0.001
Fibrosis	6	0	9	0.03	15	0	0	0.001

*P* value < 0.05.

Positive: ranks of day 8 > ranks of day 0.

Negative: ranks of day 8 < ranks of day 0.

Equal: ranks of day 8 is equal to ranks of day 0.
